# Acceptance of Deprescribing Recommendations for Fall Risk Increasing Medications

**DOI:** 10.1093/geroni/igaf122.3149

**Published:** 2025-12-31

**Authors:** Michelle Paulsen, Katherine Ritchey, Karl Brown, Erica Martinez

**Affiliations:** Department of Veterans Affairs, Seattle, Washington, United States; Department of Veterans Affairs, Seattle, Washington, United States; VA Puget Sound Health Care System, Seattle, Washington, United States; Department of Veterans Affairs, Seattle, Washington, United States

## Abstract

Fall risk increasing medications (FRIMs) have negative effects on balance and psychomotor functioning which increase the risk of falling in older adults. The Drug Burden Index (DBI) measures total exposure to anticholinergic and sedative medications and has been associated with falling risk in older adults. We evaluated whether reporting DBI score would influence deprescribing of FRIMs in older veterans at risk of falling. From January 2020 to September 2024, a pharmacist conducted a medication review for FRIMs in older veterans at risk of falling. The primary care prescriber (PCP) was alerted to recommendations through the electronic medical record. DBI was reported to PCP in a subset of patients. Changes to FRIMs and DBI score were assessed for all participants three months after initial medication review. Across the 112 study participants, the mean age was 75, 93 (83%) were male, and 89 (80%) were White. Medication-related recommendations were given to 72 (64%) of the 112 participants, with a mean (SD) of 1.9 (1.2) recommendations per patient. Of these, 37 (51%) had a recommendation accepted. The DBI was shared with care providers for 47 (65%) of the 72 who received a recommendation. Recommendation acceptance was higher in the group that did not have their DBI shared (17/25, 68%) compared to the group that did have their DBI shared (20/47, 43%). Though pharmacy intervention led to a reduction in FRIMs, DBI score did not appear to increase the acceptance of FRIM deprescribing recommendations by PCP.

